# Supplementary material for: The Drivers of Mesozoic Neoselachian Success and Resilience
Source: Biology (Basel). Author manuscript; Available in PMC 2025 Feb 26. (PMC11852107; doi:10.3390/biology14020142)
Supplement: 1 [file EMS203524-supplement-1.pdf]

Supplementary Data:

Table S1: Included neoselachian genera with their respective first and last appearance date in Ma

Genus	FAD (Ma)	LAD (Ma)
Abdounia	72,1	27,82
Acanthoscylli	93,9	83,5
Acrolamna	100,5	66
Acrosqualiolu	47,8	37,8
Actinobatis	85,8	83,5
Acutalamna	100,5	93,5
Adnetoscylliu	100,5	70,6
Aetobatus	58,7	0
Aetomylaeus	56	0
Agaleorhynch	85,8	70,6
Agaleus	196,5	174,1
Akaimia	166,1	157,3
Aktaua	48,6	37,2
Almascyllium	99,6	83,5
Alopias	56	0
Altusmirus	139,8	132,6
Amamriabatis	41,3	38
Amblyraja	5,333	0
Angolabatis	83,5	66
Angoumeius	47,8	38
Ankistrorhync	89,8	66
Annea	182,7	93,5
Anomotodon	132,9	15,97
Anoxypristis	83,5	0
Antiquaobatis	189,6	183
Antrigoulia	140,2	132,6
Apocopodon	100,5	11,62
Aprionodon	47,8	5,33
Apristurus	56	0
Aquilolamna	93,5	89,3
Araloselachus	100,5	0,01
Archaeogaleu	140,2	132,6
Archaeolamn	125	66
Archaeomant	66	33,9
Archaeotriakis	83,5	66
Archingeayia	99,6	93,5
Arechia	56	38
Asflapristis	93,5	89,3
Asterodermus	157,3	145
Ataktobatis	83,5	66
Atlanticoprist	100,5	93,9
Atlantoraja	33,9	0
Aturobatis	56	33,9
Baharipristis	99,6	93,5
Bathyraja	56	0
Bathytoshia	20,44	0

Bavariscylliur	157,3	145
Belemnobatis	182,7	113
Beringraja	7,25	0
Biropristis	83,6	66
Borodinoprist	85,8	70,6
Brachaelurus	140,2	0
Brachycarcha	66	33,9
Breviacanthus	168,3	164,7
Britobatos	93,9	83,5
Burnhamia	61,6	15,97
Bythaelurus	33,9	0
Cadiera	140,2	132,6
Cantioscylliur	157,3	66
Carcharhinus	113	0
Carcharias	145	0
Carcharodon	100,5	0
Carcharoides	72,1	2,59
Cardabiodon	100,5	83,6
Casieria	66	37,2
Caucasochas	33,9	28,4
Cederstroemi	125	70,6
Celtipristis	130	125
Cenocarchari	100,5	66
Centrophoroi	100,5	66
Centrophorus	83,5	0
Centroscymn	136,4	0
Centroselach	66	0
Centrosqualu	85,8	66
Cestracion	113	66
Cetorhinus	56	0
Chaenogaleu	58,7	0
Chiloscyllium	182,7	0
Chlamydosela	86,3	0
Coelometlaou	56	41,2
Columbusia	85,8	70,6
Corysodon	157,3	113
Cosmopolitoc	33,9	3,6
Coupatezia	83,5	33,9
Crassescylior	85,8	33,9
Crassodontid	190,8	152,1
Cretalamna	130	11,62
Cretaplatyrhir	85,8	70,6
Cretascyliorh	113	86,3
Cretascymnu	85,8	66
Cretasquatina	83,6	66
Cretodus	125	66
Cretomanta	100,5	66

Cretorectolob	136,4	66
Cretoxyrhina	152,1	66
Cristabatis	182,7	174,1
Cristomylus	99,6	66
Ctenopristis	85,8	61,6
Cyclobatis	100,5	93,5
Dalatias	208,5	0
Dallasiella	100,5	89,3
Dalpiazia	83,5	66
Danogaleus	70,6	61,6
Dasyatis	145	0
Dasyrhomboc	70,6	66
Deania	83,5	0
Delpitia	66	56
Delpitoscylliu	66	56
Diprosopover	99,6	93,5
Dipturus	33,9	0
Doliobatis	182,7	174,1
Dorsetoscylliu	168,3	166,1
Duffinselache	208,5	201,3
Dwardius	125	83,5
Dykeius	83,5	70,6
Echinorhinus	140,2	0
Enantiobatis	99,6	93,5
Engaibatis	152,1	145
Engolismaia	100,5	93,5
Eodalatias	56	47,8
Eoetmopterus	99,6	66
Eogaleus	56	47,8
Eometlaouia	61,6	41,3
Eomobula	56	41,3
Eoplatyrhina	56	47,8
Eoplinthicus	41,3	33,9
Eoptolamna	130	70,6
Eorhinobatos	56	47,8
Eorhinoptera	56	47,8
Eoscymnus	47,8	41,2
Eosqualiolus	47,8	15,97
Eostegostoma	85,8	33,9
Eostriatolami	113	66
Eotorpedo	66	41,3
Erguitaia	85,8	66
Etmopterus	48,6	0
Eugaleus	56	27,82
Eypea	168,3	164,7
Florenceodon	83,5	70,6
Fontitrygon	7,25	0

Fornicatus	139,8	132,6
Foumtizia	70,6	33,9
Fredipristis	70,6	66
Galeocerdo	59,2	0
Galeocorax	100,5	66
Galeorhinus	100,5	0
Galeus	55,8	0
Ganntouria	70,6	61,6
Ganopristis	85,8	66
Garabatis	56	33,9
Garrigascylliu	140,2	132,6
Gibbechinorh	72,1	66
Ginglymoston	100,5	0
Glikmania	48,6	37,2
Glueckmanot	59,2	47,8
Glyphis	23,03	0
Grozonodon	228	208,5
Gryphodobati	23,03	5,33
Gymnura	99,6	0
Haimirichia	100,5	89,8
Hamrabatis	100,5	66
Hemipristis	56	0
Hemiscyllium	113	0
Heptranchias	72,1	0
Hessinodon	83,5	70,6
Heterobatis	61,6	56
Heterodontus	182,7	0
Heterophorcy	168,3	166,1
Heterotorped	70,6	41,3
Hexanchus	125	0
Hexatrygon	47,8	0
Himantura	47,8	0
Hispidaspis	125	83,6
Hologinglymo	61,6	56
Hueneichthys	208,5	201,3
Hypogaleus	37,8	0
Hypolophites	66	56
Hypolophodo	86,3	15,97
Hypotodus	83,5	11,62
Hypsobatis	83,5	66
Iago	41,2	0
Iansan	122,46	112,03
Igdabatis	83,6	66
Ikamauius	28,1	3
Ischyrhiza	100,5	7,246
Ishaquia	66	56
Isistius	70,6	0

Isurolamna	61,6	27,82
Isurus	145	0
Ixobatis	70,6	61,6
Jacquherman	59,2	33,9
Jaekelotodus	72,1	37,8
Johnlongia	125,45	83,5
Jurobatos	182,7	170,3
Kallodentis	56	33,9
Keasius	48,6	11,62
Keichouodus	235	232
Kenolamna	100,5	93,5
Khouribgaleu:	61,6	56
Kiestus	99,6	89,3
Kimmerobatis	157,3	145
Komoksodon	83,5	70,6
Lamiostoma	48,6	37,2
Lamna	182,7	0
Leidybatis	72,1	33,9
Leptocharias	86,3	0
Leptostyrax	125	66
Lessiniabatis	56	47,8
Lethenia	33,9	27,82
Libanopristis	100,5	93,5
Lophobatis	56	33,9
Macrorhizodu	72,1	27,82
Macrourogale	157,3	145
Maculabatis	47,8	41,2
Mafdetia	99,6	93,5
Magistrauia	140,2	132,6
Marambioraja	56	47,8
Marckgrafia	113	93,5
Mecotrygon	41,3	38
Megachasma	28,1	0
Megalolamna	23,03	5,33
Megascyliorhi	72,1	0,34
Megasqualus	66	2,59
Mennerotodu	47,8	38
Merabatis	56	41,3
Meridiania	56	38
Meridiogaleus	56	33,9
Mesetaraja	56	47,8
Mesiteia	182,7	47,8
Microcarchari	100,5	86,3
Microetmopte	70,6	66
Micropristis	99,6	89,3
Microscyliorh	61,6	33,9
Microtoxodus	182,7	174,1

Miroscyllium	20,44	15,97
Misrichthys	48,6	33,9
Mitsukurina	55,8	0
Mobula	47,8	0
Moerigaleus	41,3	33,9
Mucrovenator	247,2	242
Mustelus	59,2	0
Myledaphus	93,5	61,6
Myliobatis	83,6	0
Myliodasyatis	66	61,6
Nanocetorhin	20,44	15,97
Nanocorax	100,5	70,6
Narcine	56	0
Nebriimimus	5,33	3,6
Nebrius	72,1	0
Negaprion	56	0
Nemacanthus	268,8	166,1
Neotrygon	38	0
Notidanodon	136,4	47,8
Notidanoides	189,6	145
Notidanus	145	3,6
Notoramphos	56	41,2
Notorhynchus	100,5	0
Occitanodus	139,8	132,6
Odontaspis	140,2	0
Odontorhytis	56	33,9
Oligodalatias	33,9	28,1
Onchopristis	145	33,9
Onchosaurus	100,5	47,8
Orectoloboide	125	37,8
Orectolobus	59,2	0
Ornatoscylliu	180,1	161,2
Orpodon	61,6	59,2
Orthechinorh	56	28,1
Ostarriraja	20,44	15,97
Ostenoselach	196,5	189,6
Otodus	66	0
Ouledia	59,2	33,9
Oxynotus	5,33	0
Pachygaleus	59,2	37,2
Pachygymnur	37,8	33,9
Pachyhexanc	140,2	129,4
Pachyscylliun	37,2	3,6
Palaeoanacor	100,5	89,3
Palaeobracha	182,7	125
Palaeocarcha	168,3	145
Palaeocarcha	66	33,9

Palaeocentro	20,44	15,97
Palaeodasyat	66	61,6
Palaeogaleus	89,8	47,8
Palaeohypoto	83,6	19
Palaeorectolc	157,3	145
Palaeorhinco	175,6	33,9
Palaeoscylliu	168,3	72,1
Palaeospinax	208,5	157,3
Palaeotriakis	86,3	70,6
Paleogenotod	56	47,8
Palidiplospina	201,3	174,1
Paracestracic	182	132,6
Paraechinorh	47,8	5,333
Paraetmopter	56	37,8
Paragaleus	56	0
Paraginglymo	175,6	113
Parahemiscyl	140,2	132,6
Paraisurus	125	66
Paranomotod	113	66
Paranotidanu	168,3	166,1
Paraorthacod	201,3	33,9
Parapalaeoba	93,5	66
Pararaja	99,6	93,5
Pararhincodo	113	33,9
Parasquatina	93,9	66
Paratriakis	100,5	66
Paratrygon	37,2	0
Paratrygonorr	70,6	66
Parotodus	48,6	2,59
Pastinachus	38	0
Peyeria	100,5	93,5
Phorcynis	163,5	145
Physogaleus	61,6	4,75
Platyrhina	56	0
Platyrhinoidis	47,8	0
Platyrhizodon	86,3	70,6
Platyrhizoscyl	47,8	38
Plesiozanoba	56	47,8
Plicatolamna	113	66
Plicatopristis	83,5	66
Plicatoscylliu	85,8	38
Plinthicus	33,9	5,33
Pliotrema	59,2	56
Porodermoide	66	56
Posadaia	100,5	89,3
Potamotrygor	48,6	0
Potobatis	66	61,1

Praeproscylliu	168,3	166,1
Premontreia	66	4,9
Prionace	23,03	0
Pristiophorus	85,8	0
Pristis	161,2	0
Procestration	157,3	145
Proetmopteru	70,6	66
Prohaploblep	89,8	70,6
Proheterodon	168,3	164,7
Promyliobatis	56	47,8
Propristiopho	72,1	66
Propristis	55,8	33,9
Prosopodon	70,6	61,6
Protocentropl	100,5	70,6
Protoginglymo	56	37,2
Protoheptrano	83,5	70,6
Protoheterodo	83,6	66
Protohimantu	23,03	15,97
Protolamna	140,2	66
Protoplatyrhir	93,5	66
Protoscyliorhi	130	86,3
Protospinax	182,7	100,5
Protosqualus	130	66
Protoxynotus	93,5	66
Pseudabdour	56	38
Pseudaetobat	56	33,9
Pseudobatos	59,2	56
Pseudocarcha	55,8	0
Pseudocetorh	208,5	201,3
Pseudocorax	100,5	66
Pseudodalatia	228	201,3
Pseudodontas	83,5	66
Pseudoechino	72,1	55,8
Pseudogingly	70,6	38
Pseudohypolo	130	66
Pseudomegac	105,3	89,3
Pseudomyled	93,5	66
Pseudonotida	183	157,3
Pseudoplatyrl	85,8	70,6
Pseudorhina	163,5	132,6
Pseudorhinob	56	47,8
Pseudoscapa	113	86,3
Pseudoscylio	100,5	70,6
Pseudospinax	166,1	93,5
Pteromylaeus	23,03	0
Pteroplatytryg	20,44	0
Pteroscyllium	125,45	61,6

Ptychodus	145	61,6
Ptychotrygon	100,5	48,6
Ptychotrygono	100,5	89,3
Pucabatis	83,6	61,6
Pucapristis	72,1	47,8
Pueblocarcha	85,8	83,5
Raineria Raja	208,5	201,3
Reifia	85,8	0
Renpetia	221,5	215,56
Restesia	99,6	93,5
Rhaibodus	83,5	66
Rhincodon	38	33,9
Rhinobatos	28,1	0
Rhinoptera	152,1	0
Rhinoscymnu	100,5	0
Rhizoprionodo	83,5	0
Rhombodus	72,1	0
Rhomboptery	85,8	56
Rhomphaiodo	99,6	93,5
Rhynchobatus	228	174,1
Rolfodon	85,8	0
Roulletia	89,8	7,246
Saltirius	100,5	93,5
Scapanorhync	40,4	37,2
Schizorhiza	182,7	11,62
Scindocorax	83,6	56
Sclerorhynch	85,8	83,5
u Scoliodon	100,5	56
Scyliorhinothe	56	0
Scyliorhinus	38	33,9
Scymnodalati	139,8	0
Scymnodon	47,8	0
Serratolamna	23,03	0
Sigmoscyllium	100,5	33,9
Similiteroscyl	93,9	70,6
Smithraja	166,1	132,6
Somniosus	56	47,8
Spathobatis	41,2	0
Sphenodus	168,3	125
Sphyrna	208,5	38
Squalicorax	58,7	0
Squaliodalatia	145	56
Squaliolus	100,5	15,97
Squalogaleus	47,8	0
Squalus	164,7	161,2
Squatigaleus	125	0
	83,5	66

Squatina	163,5	0
Squatirhina	125,45	66
Squatiscyllium	83,5	41,2
Stegostoma	47,8	0
Stenoscyllium	56	41,3
Striatolamia	83,6	4,3
Subathunura	58,7	47,8
Sulcidens	61,6	59,2
Sylvestrilamia	56	41,3
Synechodus	295,5	41,2
Synodontaspi	56	23,03
Taeniura	11,62	0
Taeniurops	33,9	0
Tanoutia	70,6	66
Telodontaspis	99,6	89,3
Tethybatis	83,5	66
Tethylamna	56	38
Tethytrygon	56	47,8
Texabatis	70,6	66
Texatrygon	93,9	70,6
Thiesus	167,7	132,6
Tingitanius	100,5	89,3
Titanonarke	56	47,8
Tlalocbatos	113	99,6
Toarcibatis	182,7	174,1
Tomewingia	72,1	66
Torpedo	56	0
Triaenodon	47,8	0
Triakis	70,6	0
Trigonognathu	47,8	0
Trigonotodus	41,2	37,8
Truyolsodonto	100,5	93,5
Turoniabatis	125	86,3
Urobatis	56	0
Urolophus	56	0
Vallisodus	208,5	201,3
Vascobatis	70,6	66
Viperecucullu	66	61,7
Walteraja	70,6	66
Weissobatis	33,9	27,82
Welcommia	140,2	132,6
Weltonia	66	47,8
Woellsteinia	66	38
Xampylodon	125	66
Xiphodolamia	59,2	5,33
Xystrogaleus	47,8	38
Youssoubatis	83,5	61,6

Zameus	5,33	0
Zapteryx	28,1	0

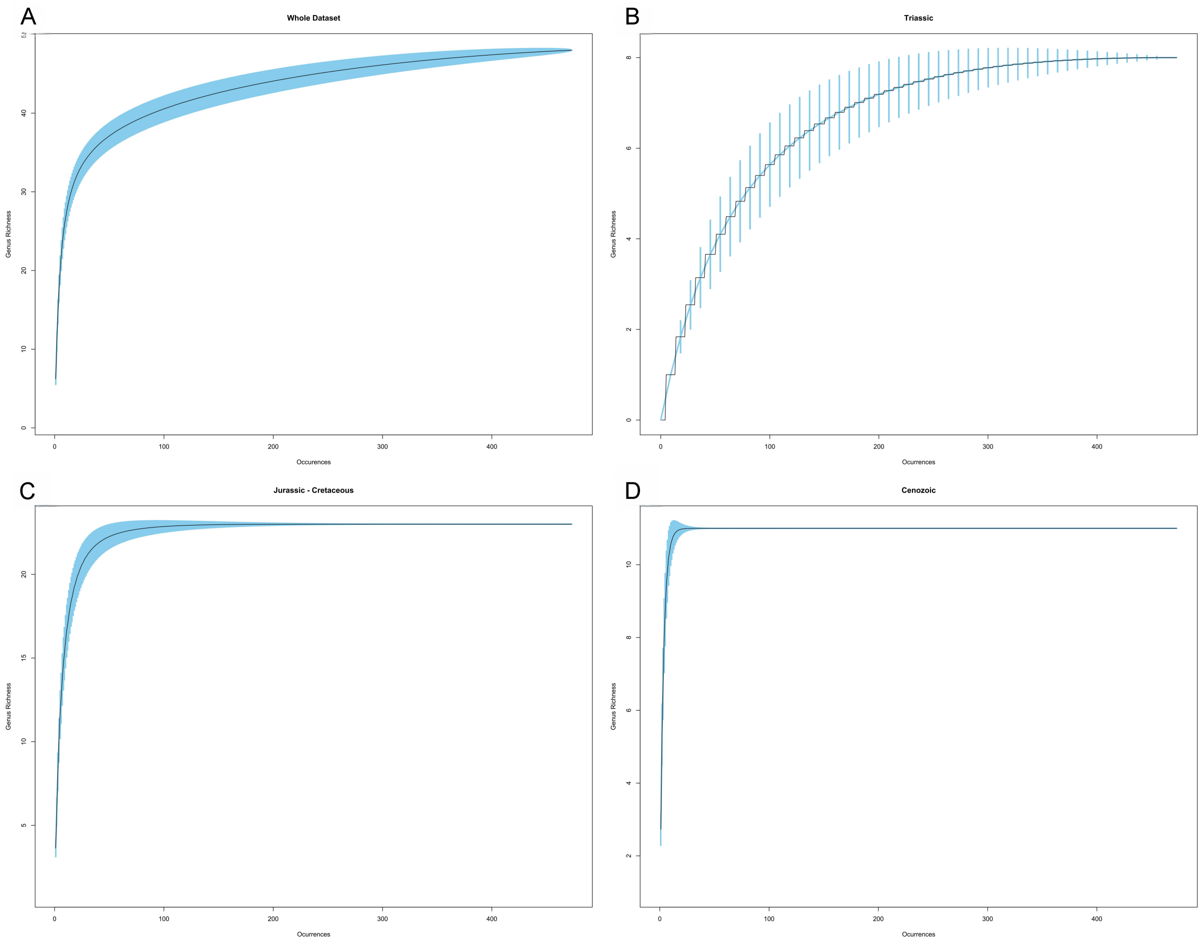

Figure S1: Rarefaction curves of genus richness (CI = 0.95) per period bin for neoselachian fossil occurrences. Blue bars/ shading shows the 95% confidence intervals (1000 permutations) (A) Whole dataset, (B) Triassic, (C) Jurassic + Cretaceous, (D) Cenozoic;

Table S2: Detrended correspondence analysis results for neoselachian fossil occurrences for the Mesozoic and Cenozoic. Values are included from bin 11 (mid Ma = 287) to bin 58 for all four axes. Eigenvalues, additive eigenvalues, decorona values and axis length are additionally included below the individual DCA scores.

Mid Ma	Time Bin	DCA1	DCA2	DCA3	DCA4
287	11	5,92343086	2,18583841	-4,51E-06	-4,9834705
281	12	5,92343086	2,18583841	-4,51E-06	-4,9834705
275	13	5,92343086	2,18583841	-4,51E-06	-4,9834705
269	14	6,67374923	2,22299919	-1,09E-06	-0,8154365
263	15	6,67374923	2,22299919	-1,09E-06	-0,8154365
257	16	6,67374923	2,22299919	-1,09E-06	-0,8154365
251	17	6,67374923	2,22299919	-1,09E-06	-0,8154365
245	18	6,92753306	2,47965261	-2,08E+00	-0,440818
239	19	6,67374923	2,22299919	-1,09E-06	-0,8154365
233	20	6,92753306	2,4796549	2,08E+00	-0,440818
227	21	7,87456773	0,51336507	2,10E-06	-0,7498184
221	22	9,64977595	0,62486163	1,61E-06	-0,6752412
215	23	9,64977595	0,62486163	1,61E-06	-0,6752412
209	24	7,36740684	-2,1464079	-2,39E-06	0,02391596
203	25	6,77948852	-2,1548569	-1,36E-06	0,08847188
197	26	6,42766206	-0,8528437	-5,79E-08	0,04855124
191	27	6,37670397	-0,9359312	1,08E-06	-0,1572722
185	28	4,8601343	-0,628493	3,48E-06	-1,3299786
179	29	4,66025687	-0,6281849	3,28E-06	-1,1902081
173	30	4,42266937	-0,5989324	2,99E-06	-1,1437439
167	31	4,01201197	-0,5255106	2,08E-07	4,4190976
161	32	3,72613871	-0,4069232	-7,32E-07	0,82415314
155	33	3,54775695	-0,3047538	-3,76E-06	-0,8153364
149	34	3,26000182	-0,218936	-4,47E-06	-1,1365881
143	35	2,65937964	-0,1100705	-4,65E-06	-2,445257
137	36	2,33286877	-0,0022694	-3,14E-06	-3,8235872
131	37	2,26214364	0,02012473	-1,87E-06	-2,1521295
125	38	1,97189657	0,04784815	6,28E-09	-0,7368936
119	39	1,91257506	0,05761932	-1,99E-08	-0,7146825
113	40	1,81774272	0,07964409	5,96E-07	-0,443362
107	41	1,71797429	0,09164255	-3,32E-07	-0,3363165
101	42	1,26176546	0,17095114	-4,71E-08	2,05430766
95	43	1,14819118	0,17705169	-2,79E-07	2,90754356
89	44	0,90423921	0,14406812	-1,95E-07	1,38546553
83	45	0,31580889	0,1032128	1,57E-06	0,92169144
77	46	0,28139631	0,09529258	1,86E-06	0,77712329
71	47	0,01274485	0,08515665	3,36E-06	0,59788179
65	48	-0,1605731	0,06512267	3,79E-06	0,19416726
59	49	-0,4751158	0,01168301	7,57E-07	-0,4299294
53	50	-0,9047287	-0,0109546	-7,74E-07	-0,969844
47	51	-1,2187046	-0,0116431	-3,88E-07	-0,9737644
41	52	-1,3328047	-0,0054847	5,88E-08	-0,7761831
35	53	-2,0350141	-0,0101623	-1,67E-06	-0,4263737
29	54	-2,3307875	0,00199358	-1,90E-06	-0,0087142
23	55	-2,883812	0,00697372	-7,69E-07	0,10414363
17	56	-2,8267093	0,00715315	-1,17E-06	0,11544727

11	57	-2,6307115	0,00543846	-1,06E-06	0,08568521
5	58	-4,4423403	0,00777107	-9,92E-07	0,05451975

Detrended correspondence analysis with 26 segments.
Rescaling of axes with 6 iterations.
Total inertia (scaled Chi-square): 5.5246

	DCA1	DCA2	DCA3	DCA4
Eigenvalues	0.6922	0.4563	0.3333	0.2383
Additive Eigenvalues	0.6922	0.4470	0.3333	0.2424
Decorana values	0.7483	0.3911	0.3333	0.2058
Axis lengths	14.1366	5.8563	4.1450	4.2682

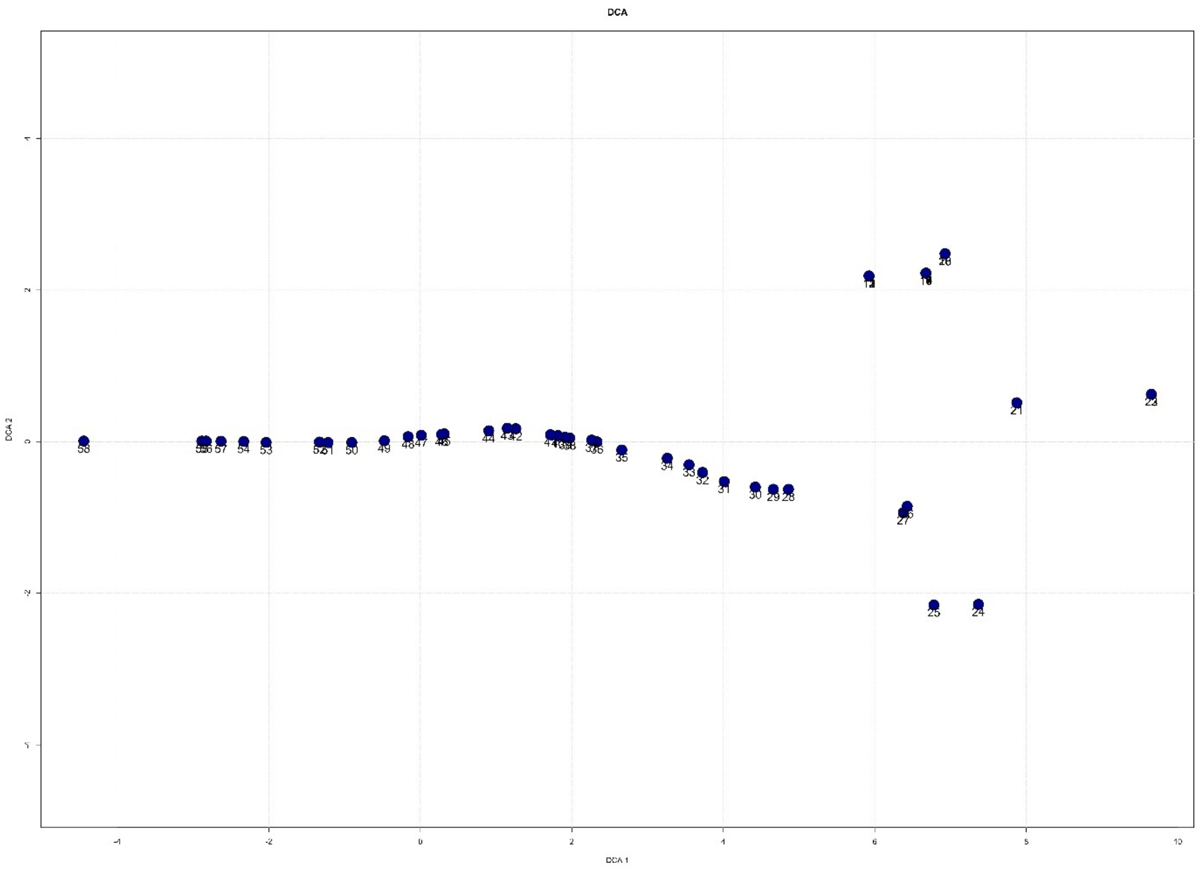

Figure S2: Changes in neoselachian faunal composition shown by scores of samples on DCA axis 1 against axis 2. Blue dots represent the position of the fauna of a specific bin within the ordination space. Units of the x- and y axis are standard deviation as a metric for faunal turnover. Numbers next to the dot indicate the number of the respective time bin.

Table S3: Used time bins including their upper and lower boundary, their mid Ma and bin number.

bottom Ma	mid Ma	top Ma	Bin #
350	347	344	1
344	341	338	2
338	335	332	3
332	329	326	4
326	323	320	5
320	317	314	6
314	311	308	7
308	305	302	8
302	299	296	9
296	293	290	10
290	287	284	11
284	281	278	12
278	275	272	13
272	269	266	14
266	263	260	15
260	257	254	16
254	251	248	17
248	245	242	18
242	239	236	19
236	233	230	20
230	227	224	21
224	221	218	22
218	215	212	23
212	209	206	24
206	203	200	25
200	197	194	26
194	191	188	27
188	185	182	28
182	179	176	29
176	173	170	30
170	167	164	31
164	161	158	32
158	155	152	33
152	149	146	34
146	143	140	35
140	137	134	36
134	131	128	37
128	125	122	38
122	119	116	39
116	113	110	40
110	107	104	41
104	101	98	42
98	95	92	43
92	89	86	44
86	83	80	45
80	77	74	46

74	71	68	47
68	65	62	48
62	59	56	49
56	53	50	50
50	47	44	51
44	41	38	52
38	35	32	53
32	29	26	54
26	23	20	55
20	17	14	56
14	11	8	57
8	5	2	58

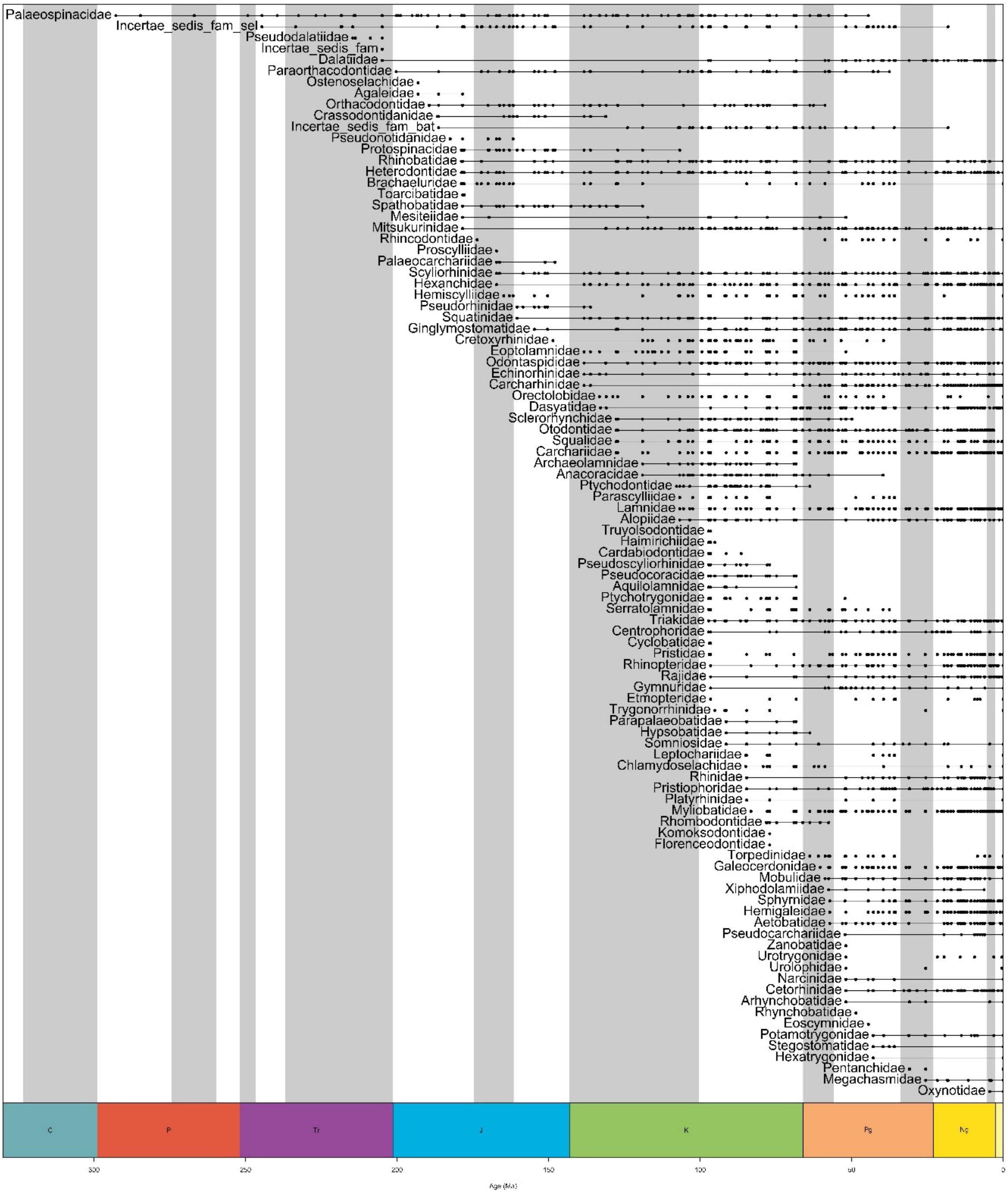

Figure S3: Chronostratigraphic range chart of neoselachian family fossil occurrences. Solid black dots represent one or multiple fossil occurrences at the respective time of the respective family. Solid black bars represent the entire period between first and last known occurrence of the individual family.

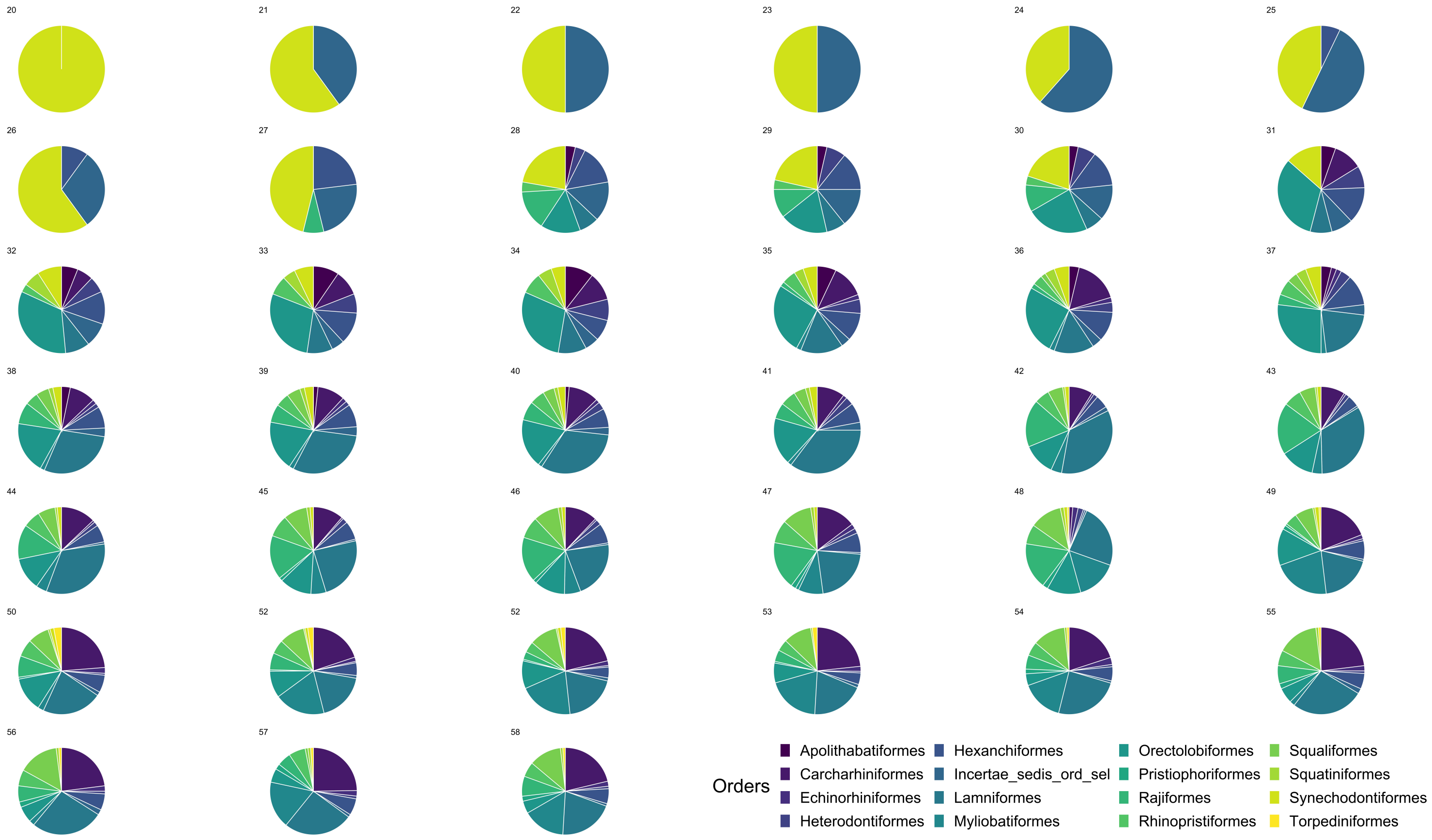

Figure S4: Relative faunal composition of neoselachians through time. Pie charts show the faunal composition of the indicated bin in terms of genus richness of the individual orders. Displayed pie charts start with bin 20, as all previous bins contain only one order (Synechodontiformes).

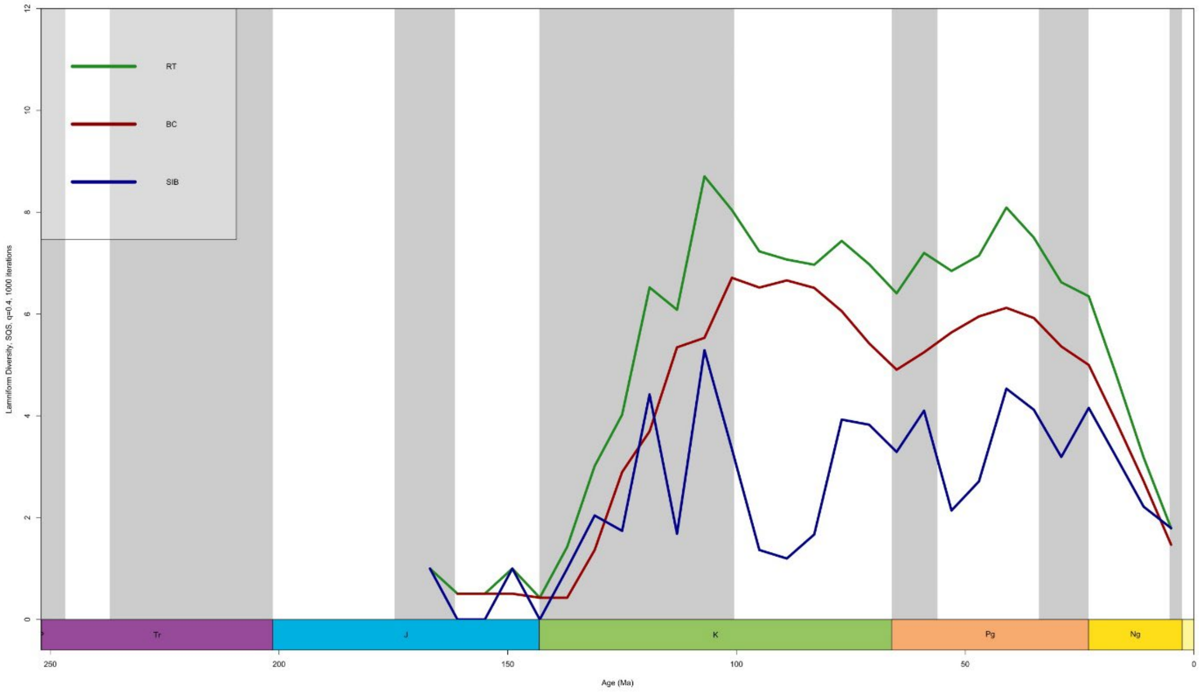

Figure S5: Diversity dynamics of lamniform shark genera through the Mesozoic and Cenozoic based on SQS data (q=0.4, 1000 iterations). The three curves represent the three used diversity approach: Sampled in bin (SIB, blue solid line), range through (RT, green solid line) and boundary crosser (BC, red solid line).

	Carboniferous								Permian								Triassic								Jurassic										Cretaceous														Palaeogene							Neo		
Apolithabatiformes	0	0	0	0	0	0	0	0	0	0	0	0	0	0	0	0	0	0	0	0	0	0	0	0	0	0	0	1	1	1	2	2	4	4	4	2	2	2	1	1	0	0	0	0	0	0	0	0	0	0	0	0	0	0	0	0	0	0
Carcharhiniformes	0	0	0	0	0	0	0	0	0	0	0	0	0	0	0	0	0	0	0	0	0	0	0	0	0	0	0	0	0	0	4	2	4	4	7	9	1	6	6	8	7	11	12	16	18	18	24	2	25	33	38	37	38	23	24	23	24	23
Echinorhiniformes	0	0	0	0	0	0	0	0	0	0	0	0	0	0	0	0	0	0	0	0	0	0	0	0	0	0	0	0	0	0	0	0	0	0	1	1	1	1	1	1	1	1	1	1	1	1	3	3	2	3	3	3	3	3	2	2	2	2
Heterodontiformes	0	0	0	0	0	0	0	0	0	0	0	0	0	0	0	0	0	0	0	0	0	0	0	0	0	0	0	1	2	2	3	2	3	3	3	2	2	1	1	2	2	2	2	2	3	3	3	3	1	1	1	1	1	1	1	1	1	1
Hexanchiformes	0	0	0	0	0	0	0	0	0	0	0	0	0	0	0	0	0	0	0	0	0	0	0	0	1	1	3	4	4	4	5	4	5	3	6	6	6	5	5	5	5	6	6	8	11	11	12	1	9	9	9	7	7	6	6	6	6	6
Incertae_sedis_ord_sel	0	0	0	0	0	0	0	0	0	0	0	0	0	0	0	0	0	0	0	0	2	3	3	8	7	3	3	4	4	4	3	3	2	2	2	2	2	2	2	2	2	2	1	1	1	1	1	1	1	2	2	2	2	1	2	2	1	1
Lamniformes	0	0	0	0	0	0	0	0	0	0	0	0	0	0	0	0	0	0	0	0	0	0	0	0	0	0	0	2	2	2	3	3	4	4	9	8	11	18	18	23	24	44	45	41	38	32	35	36	25	31	35	34	32	28	28	27	25	22
Myliobatiformes	0	0	0	0	0	0	0	0	0	0	0	0	0	0	0	0	0	0	0	0	0	0	0	0	0	0	0	0	0	0	0	0	0	0	1	1	1	1	1	1	1	5	5	5	9	9	15	23	28	3	36	35	32	18	2	2	17	17
Orectolobiformes	0	0	0	0	0	0	0	0	0	0	0	0	0	0	0	0	0	0	0	0	0	0	0	0	0	0	0	4	5	7	12	11	12	11	15	14	14	12	11	13	12	15	17	15	19	17	2	19	18	18	19	18	12	5	6	6	5	5
Pristiophoriformes	0	0	0	0	0	0	0	0	0	0	0	0	0	0	0	0	0	0	0	0	0	0	0	0	0	0	0	0	0	0	0	0	0	0	0	0	0	0	0	0	0	0	0	0	2	2	3	3	2	1	1	1	1	2	2	2	2	2
Rajiformes	0	0	0	0	0	0	0	0	0	0	0	0	0	0	0	0	0	0	0	0	0	0	0	0	0	0	1	4	3	3	0	0	0	0	1	1	2	5	4	5	4	22	26	16	26	25	29	26	1	11	12	5	7	6	7	6	5	8
Rhinopristiformes	0	0	0	0	0	0	0	0	0	0	0	0	0	0	0	0	0	0	0	0	0	0	0	0	0	0	0	1	1	1	0	1	3	3	3	2	3	3	3	4	4	7	9	8	13	12	14	11	6	9	10	7	7	6	6	6	6	6
Squaliformes	0	0	0	0	0	0	0	0	0	0	0	0	0	0	0	0	0	0	0	0	0	0	0	0	0	0	0	0	0	0	0	0	0	0	0	1	2	3	3	3	3	7	8	8	14	14	18	18	9	11	18	18	17	14	16	15	1	13
Squatiniformes	0	0	0	0	0	0	0	0	0	0	0	0	0	0	0	0	0	0	0	0	0	0	0	0	0	0	0	0	0	0	0	2	2	2	2	2	2	1	1	1	1	1	1	1	2	2	2	2	1	1	1	1	1	1	1	1	1	1
Synechodontiformes	0	0	0	0	0	0	0	0	0	0	1	1	1	2	2	2	2	3	2	3	3	3	3	5	6	6	6	6	6	6	5	3	3	2	3	3	3	2	2	2	2	2	2	2	2	2	2	2	2	2	2	2	0	0	0	0	0	0
Torpediniformes	0	0	0	0	0	0	0	0	0	0	0	0	0	0	0	0	0	0	0	0	0	0	0	0	0	0	0	0	0	0	0	0	0	0	0	0	0	0	0	0	0	0	0	0	0	0	0	1	1	4	4	3	3	1	1	1	1	1

Figure S6: Heatmap of raw neoselachian genus diversity per order in the respective time bin. Green tiles indicate a lack of diversity, light yellow tiles a low standing diversity with an increase to red shades with high standing diversity in the corresponding order in the respective bin (bins equal 6 Ma each).

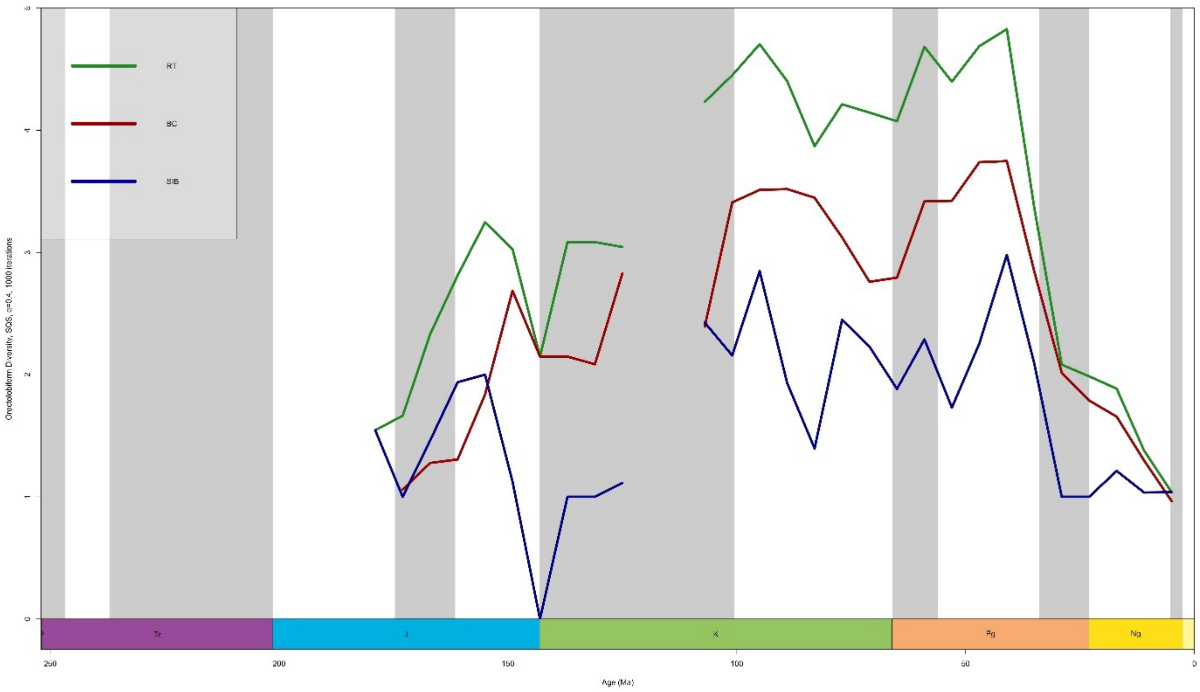

Figure S7: Diversity dynamics of orectolobiform shark genera through the Mesozoic and Cenozoic based on SQS data (q=0.4, 1000 iterations). The three curves represent the three used diversity approach: Sampled in bin (SIB, blue solid line), range through (RT, green solid line) and boundary crosser (BC, red solid line). The gap in the curve in the Early Cretaceous is results from the sample standardisation due to uneven sampling of the taxon in the Early Cretaceous.

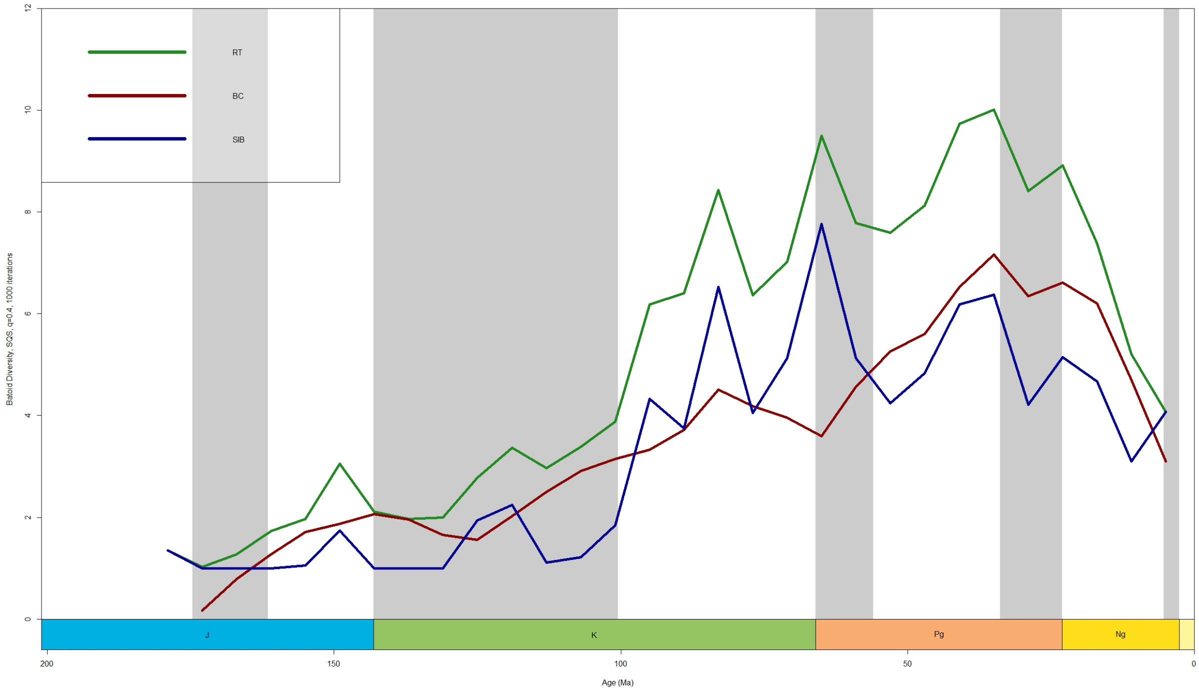

Figure S8: Diversity dynamics of batoid genera through the Mesozoic and Cenozoic based on SQS data (q=0.4, 1000 iterations). The three curves represent the three used diversity approach: Sampled in bin (SIB, blue solid line), range through (RT, green solid line) and boundary crosser (BC, red solid line). The late onset of he curve in the Early Jurassic is results from the sample standardisation due to low fossile occurences and diversity during this period.

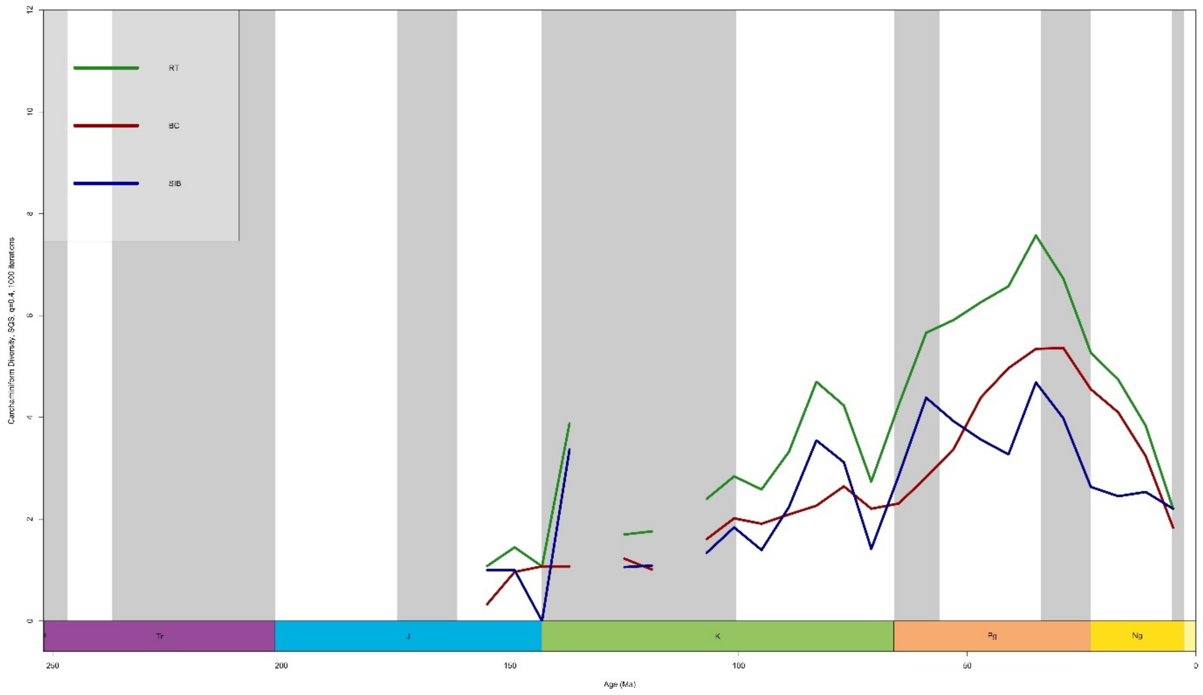

Figure S9: Diversity dynamics of carcharhiniform shark genera through the Mesozoic and Cenozoic based on SQS data (q=0.4, 1000 iterations). The three curves represent the three used diversity approach: Sampled in bin (SIB, blue solid line), range through (RT, green solid line) and boundary crosser (BC, red solid line). The two Early Cretaceous discontinuities and the late onset of the curves are caused by the sample standardisation due to uneven sampling of the taxon in the Early Cretaceous and low diversity and occurences in the Middle to Late Jurassic.

Table S4: Results of the Spearman's rank correlation analysis per taxonomic unit and diversity approach (SQS) for biotic and abiotic predictors.

Variable 1	Variable 2	Rho	p-Value (95% CI)
Neo SIB	Neo RT	0,6956522	2,353E-04
Neo BC	Neo RT	0,953913	1,784E-06
Neo SIB	Neo BC	0,5234783	9,548E-03
Neo DCA	Neo SIB	-0,5226087	9,685E-03
Neo DCA	Neo RT	-0,9573913	1,700E-06
Neo DCA	Neo BC	-0,9817391	1,118E-06
Sel SIB	Bat SIB	0,220972	3,492E-01
Sel RT	Bat RT	0,8586466	2,200E-16
Sel BC	Bat BC	0,8859649	2,200E-16
Neo SIB	SL	0,3652174	8,000E-02
	SST	-0,2913043	1,669E-01
	aCO2	-0,2104348	3,221E-01
	Flood	0,3626087	8,230E-02
	FragInd	0,4713043	2,121E-02
	Dino	0,6330435	1,161E-03
	Bony	0,5130435	1,130E-02
	Nanno	0,3886957	6,144E-02
	Foram	0,4124036	8,899E-02
Sel SIB	SL	0,3286957	1,171E-01
	SST	-0,26	2,189E-01
	aCO2	-0,2026087	3,408E-01
	Flood	0,3513043	9,287E-02
	FragInd	0,4443478	3,076E-02
	Dino	0,5991304	2,404E-03
	Bony	0,4713043	2,121E-02
	Nanno	0,386087	6,332E-02
	Foram	0,3968997	1,029E-01
Bat SIB	SL	0,7353035	2,208E-04
	SST	0,111248	6,405E-01
	aCO2	-0,6156738	3,853E-03
	Flood	0,6004343	5,122E-03
	FragInd	0,803881	1,952E-05
	Dino	0,6202456	3,528E-03
	Bony	0,7794979	5,090E-05
	Nanno	0,4396581	5,241E-02
	Foram	0,8564306	5,727E-06
Neo RT	SL	0,8573913	2,312E-06
	SST	-0,013913	9,497E-01
	aCO2	-0,5547826	5,592E-03
	Flood	0,826087	2,159E-06
	FragInd	0,9295652	2,323E-06
	Dino	0,9495652	1,887E-06
	Bony	0,8521739	2,222E-06
	Nanno	0,8069565	3,502E-06
	Foram	0,9002595	3,587E-07
Sel RT	SL	0,8695652	2,516E-06
	SST	0,03304348	8,788E-01
	aCO2	-0,5626087	4,855E-03
	Flood	0,8304348	2,080E-06
	FragInd	0,9330435	2,255E-06
	Dino	0,9347826	2,219E-06
	Bony	0,8243478	2,208E-06
	Nanno	0,8147826	2,706E-06
	Foram	0,9281665	2,841E-08
Bat RT	SL	0,843609	2,200E-16
	SST	-0,0691729	7,722E-01
	aCO2	-0,6330827	3,390E-03

	Flood	0,6857143	1,158E-03
	FragInd	0,9323308	5,044E-06
	Dino	0,7774436	8,087E-05
	Bony	0,8195489	6,732E-06
	Nanno	0,6270677	3,787E-03
	Foram	0,8620165	4,247E-06
Neo BC	SL	0,9191304	2,502E-06
	SST	0,04608696	8,309E-01
	aCO2	-0,6617391	5,857E-04
	Flood	0,8304348	2,080E-06
	FragInd	0,953913	1,784E-06
	Dino	0,9208696	2,476E-06
	Bony	0,8478261	2,153E-06
	Nanno	0,8182609	2,474E-06
	Foram	0,9033603	2,814E-07
Sel BC	SL	0,9504348	1,867E-06
	SST	0,1956522	3,579E-01
	aCO2	-0,6278261	1,306E-03
	Flood	0,9017391	2,683E-06
	FragInd	0,933913	2,237E-06
	Dino	0,88	2,642E-06
	Bony	0,8104348	3,096E-06
	Nanno	0,8469565	2,140E-06
	Foram	0,8630501	4,012E-06
Bat BC	SL	0,8175439	1,670E-05
	SST	-0,0122807	9,626E-01
	aCO2	-0,5719298	1,185E-02
	Flood	0,6649123	2,464E-03
	FragInd	0,922807	1,844E-06
	Dino	0,7614035	2,321E-04
	Bony	0,7508772	3,209E-04
	Nanno	0,7263158	6,294E-04
	Foram	0,8237736	2,647E-05
Neo DCA	SL	-0,9243478	2,418E-06
	SST	-0,0365217	8,660E-01
	aCO2	0,6704348	4,691E-04
	Flood	-0,8452174	2,117E-06
	FragInd	-0,9747826	1,280E-06
	Dino	-0,9321739	2,272E-06
	Bony	-0,82	2,382E-06
	Nanno	-0,8356522	2,049E-06
	Foram	-0,9519391	1,231E-09

Table S5: Statistical results of the respective best-fitting model per taxonomic unit and diversity approach (SQS) for the abiotic predictors.

Diversity Approach	Taxonomic Group	Best Model Fit	AIC	R-squared	Adjusted R-squared	Residual standard error	F-Statistic	p-value (95% CI)	Independed Variable	Estimate	St.Error	t value	Pr(>|t|)	t-value significant
Sample in Bin	Neoselachians	SIB ~ SST + FragInd	66,72	0,5347	0,4904	3,79	12,07	3,25E-04	SST	-0,697300	0,208100	3,350	3,03E-03	✓
									FragInd	39,525200	9,675600	4,085	5,30E-04	✓
Boundary Crosser	Neoselachians	BC ~ aCO2 + Flood + FragInd	7,46	0,9657	0,9606	1,08	187,90	8,13E-15	aCO2	-0,002599	0,000858	-3,028	6,65E-03	✓
									Flood	0,112700	0,032830	3,433	2,63E-03	✓
									FragInd	40,830000	6,190000	6,597	2,00E-06	✓
Range Through	Neoselachians	RT ~ SST + aCO2 + FragInd	53,07	0,8765	0,8580	2,80	47,31	2,88E-09	SST	-0,335152	0,154014	-2,176	4,17E-02	✓
									aCO2	-0,005066	0,002193	-2,310	3,17E-02	✓
									FragInd	70,854744	9,033577	7,843	1,58E-07	✓
DCA scores	Neoselachians	DCA ~ SST + aCO2 + Flood + FragInd	-28,88	0,9511	0,9408	0,50	70,10	3,57E-12	SST	0,054550	0,030610	1,782	9,07E-02	✗
									aCO2	0,001937	0,000397	4,884	1,03E-04	✓
									Flood	-0,054040	0,016880	-3,202	4,70E-03	✓
									FragInd	-10,890000	2,995000	-3,637	1,75E-03	✓
Sample in Bin	Selachians	SIB ~ SST + FragInd	51,30	0,4779	0,4282	2,75	9,61	1,09E-03	SST	-0,461100	0,150900	-3,055	6,02E-03	✓
									FragInd	25,231900	7,016900	3,596	1,70E-03	✓
Boundary Crosser	Selachians	BC ~ aCO2 + Flood + FragInd	-4,75	0,9704	0,9660	0,84	218,60	1,88E-15	aCO2	-0,001974	0,000666	-2,966	7,64E-03	✓
									Flood	0,118500	0,025460	4,656	1,52E-04	✓
									FragInd	30,860000	4,800000	6,430	2,85E-06	✓
Range Through	Selachians	RT ~ SST + aCO2 + Flood + FragInd	-30,69	0,8785	0,8529	2,13	40,69	1,88E-08	SST	-0,209585	0,130420	-1,607	1,25E-01	✗
									aCO2	-0,002902	0,001690	-1,717	1,02E-01	✗
									Flood	0,092754	0,071906	1,290	2,13E-01	✗
									FragInd	42,446404	12,760599	3,326	3,55E-03	✓
Sample in Bin	Batoids	SIB ~ SL + SST + aCO2 + FragInd	4,27	0,8107	0,7602	1,00	16,06	2,69E-05	SL	-0,015733	0,008846	-1,778	9,56E-02	✗
									SST	0,081510	0,064290	1,268	2,24E-01	✗
									aCO2	-0,004722	0,001104	-4,279	6,60E-04	✓
									FragInd	23,006652	7,395676	3,111	7,16E-03	✓
Boundary Crosser	Batoids	BC ~ aCO2 + FragInd	-30,69	0,8945	0,8813	0,42	67,84	1,53E-08	aCO2	-0,000621	0,000456	-1,363	1,92E-01	✗
									FragInd	14,548669	1,608807	9,043	1,09E-07	✓
Range Through	Batoids	RT ~ SL + SST + aCO2 + FragInd	0,56	0,8983	0,8712	0,91	33,14	2,77E-07	SL	-0,011370	0,008063	-1,410	1,79E-01	✗
									SST	0,076901	0,058600	1,312	2,09E-01	✗
									aCO2	-0,004526	0,001006	-4,499	4,24E-04	✓
									FragInd	29,098681	6,741123	4,317	6,11E-04	✓

Table S6: Statistical results of the respective best-fitting model per taxonomic unit and diversity approach (SQS) for the biotic predictors.

Diversity Approach	Taxonomic Group	Best Model Fit	AIC	R-squared	Adjusted R-squared	Residual standard error	F-Statistic	p-value	Independed Variable	Estimate	St.Error	t value	Pr(>|t|)	t-value significant
Sample in Bin	Neoselachians	SIB ~ Foram	57,54	0,3134	0,2705	4,694	7,303	1,57E-02	Foram	0,15542	0,0575	2,702	1,57E-02	✓
Boundary Crosser	Neoselachians	BC ~ Dino + Bony + Foram	5,88	0,9267	0,911	1,069	58,97	3,47E-08	Dino	0,02751	0,01313	2,095	5,49E-02	✗
									Bony	0,02646	0,00786	3,365	4,63E-03	✓
									Foram	0,10468	0,01894	5,527	7,45E-05	✓
Range Through	Neoselachians	RT ~ Foram	37,52	0,7992	0,7866	2,691	63,68	5,73E-07	Foram	0,26314	0,03298	7,98	5,73E-07	✓
DCA scores	Neoselachians	DCA ~ Dino + Foram	-33,77	0,9297	0,9204	0,363	99,22	2,25E-09	Dino	-0,01823	0,00433	-4,207	7,63E-04	✓
									Foram	-0,04355	0,00589	-7,393	2,24E-06	✓
Sample in Bin	Selachians	SIB ~ Foram	45,95	0,1979	0,1477	3,402	3,947	6,44E-02	Foram	0,08281	0,04168	1,987	6,44E-02	✗
Boundary Crosser	Selachians	BC ~ Bony + Nanno + Foram	5,66	0,901	0,8798	1,063	42,47	2,80E-07	Bony	0,02468	0,00784	3,15	7,09E-03	✓
									Nanno	0,01906	0,00968	1,97	6,90E-02	✗
									Foram	0,08568	0,01771	4,837	2,64E-04	✓
Range Through	Selachians	RT ~ Bony + Foram	25,79	0,799	0,7722	1,898	29,81	5,94E-06	Bony	0,0194	0,01357	1,43	1,73E-01	✗
									Foram	0,15093	0,02932	5,148	1,19E-04	✓
Sample in Bin	Batoids	SIB ~ Nanno + Foram	5,97	0,7613	0,7295	1,094	23,92	2,16E-05	Nanno	-0,01579	0,00966	-1,634	1,23E-01	✗
									Foram	0,1051	0,01624	6,471	1,06E-05	✓
Boundary Crosser	Batoids	BC ~ Nanno + Foram	-21,85	0,8142	0,7895	0,5054	32,88	3,29E-06	Nanno	0,01171	0,00446	2,624	1,92E-02	✓
									Foram	0,03641	0,0075	4,854	2,10E-04	✓
Range Through	Batoids	RT ~ Foram	6,72	0,8043	0,7921	1,144	65,76	4,65E-07	Foram	0,11367	0,01402	8,109	4,65E-07	✓

Table S7: Sample standardized diversity metrics, and environmental data. For explanation of terms and sources please refer to the material and methods section in the main text.

Time Bin	mid_MA	Neo SIB	Neo RT	Neo BC	Selachian SIB	Selachian BC	Batoidean SIB	Batoidean BC	Sea Level	Sea Surface Temperature	Atmospheric CO2 conc.	Flooded Area	Dinoflaggelate Div.	Bony Fish Div.	Calcaleous Nannoplankton Div.	Planktic Foraminera Div.	Fragmentation Index	Selachian RT	Batoidean RT	Neo Origination Rate	Neo Extinction Rate
25	203	1,587	2,622	1,708	1,548	1,688	-	-	-0,4128571	15,61	1521,41478	55,1223519	26,19565217	13,7391304	5	-	0,330667738	2,57	-	0,193	0,454
26	197	1,03	2,316	1,447	1,032	1,421	-	-	6,69916667	22,88	1768,39668	57,7142823	36,25714286	15,2	10	-	0,333172978	2,286	-	0,541	0,001
27	191	1,553	3,163	1,987	1,552	1,975	-	-	18,4633333	20,42	1196,40927	56,8973589	42,36746988	13,7289157	21	-	0,316052718	3,146	-	0,64	0,259
28	185	2,991	4,377	2,368	2,591	2,37	-	-	40,914	20,02	796,281512	56,149722	50,51497006	7,35329341	28,5	-	0,316666936	4,05	-	1,067	0,469
29	179	7,093	8,937	2,966	5,452	2,909	1,309	-	67,7108333	25,41	971,158725	56,9126604	59,13772455	18,8502994	29,5	-	0,316153026	7,187	1,309	3,199	0,494
30	173	5,962	9,17	5,671	5,161	5,574	1	0,179	27,785	21,07	1040,99704	65,1591888	64,35483871	3,4516129	36	-	0,31954972	8,196	1,033	2,341	0,561
31	167	6,357	11,299	7,451	6,076	6,748	1	0,796	53,6433333	25,16	974,400588	71,7220002	120,3333333	20,8095238	45,5	3,5	0,364339083	10,572	1,259	1,276	0,465
32	161	6,553	11,431	8,262	5,678	7,15	1	1,256	115,52	19,4	785,328387	76,6010946	173,2142857	18,2272727	53	3,5	0,365285496	9,933	1,718	1,528	0,92
33	155	4,37	10,584	8,87	3,526	7,442	1,045	1,706	144,6	22,415	794,424442	74,6118615	179,3157895	36,1052632	45	2,5	0,368623258	8,857	1,96	1,086	0,439
34	149	7,669	13,233	9,517	5,551	7,845	1,731	1,866	104,807143	19,01	737,871178	71,5904595	177,2195122	39,7804878	55	3	0,380930104	10,256	3,074	1,087	1,53
35	143	1	9,16	9,074	1	7,366	1	2,051	138,189444	17,45	739,426377	70,2765405	195,0487805	21,9512195	56,5	2,5	0,396888667	7,501	2,084	0,081	0,131
36	137	10,263	15,033	9,024	9,829	7,501	1	1,934	64,665	16,29	1011,83142	73,1782962	201,7368421	16,4297521	79,5	7,5	0,397791301	13,826	1,955	1,959	1,487
37	131	5,113	12,625	9,496	4,514	8,213	1	1,629	171,142857	21,25	907,288032	80,2957266	207	4,25316456	86	7,5	0,420199518	11,128	1,954	2,387	0,593
38	125	8,233	15,507	11,29	5,977	10,041	1,98	1,475	163,537778	20,74	856,754466	85,8412895	215,3902439	43,5121951	79	10,5	0,451854357	12,792	2,772	2,77	1,829
39	119	9,345	16,818	12,231	7,687	10,536	2,283	1,994	159,894444	22,68	1085,25809	89,5323211	211	64	75	28	0,435686482	14,239	3,384	2,971	1,223
40	113	6,424	16,82	13,979	4,959	12,106	1,106	2,517	159,1	20,3025	1194,13423	85,5225637	217,8571429	71,8367347	91	26	0,461759614	14,157	2,992	1,522	0,549
41	107	11,012	20,495	14,952	9,711	12,711	1,201	2,94	184,718	20,9	1028,19837	86,9260843	224,7142857	79,6734694	105,5	19,5	0,499793016	17,658	3,401	3,305	1,938
42	101	8,585	19,787	16,319	7,457	14,126	1,832	3,173	234,65	24,66	944,649125	93,7428829	219,5811518	154,057592	111,5	30	0,50722461	17,163	3,862	1,586	1,872
43	95	1,885	17,021	16,033	1,693	13,987	4,263	3,382	221,570588	30,03	596,577119	92,3359015	211,4766355	143,785047	99	31,5	0,51137412	14,823	6,16	0,453	0,281
44	89	1,38	16,796	16,205	1,228	14,145	3,817	3,74	236,957143	29,71	477,67319	91,3608868	206,6601942	30,5	108,5	39,5	0,514559122	14,64	6,44	0,296	0,203
45	83	3,464	17,719	16,298	2,913	14,204	6,573	4,443	210,4	24,85	680,941234	100,769486	216,7464789	78,016129	122	39	0,529229458	15,307	8,417	0,619	0,851
46	77	16,589	25,447	16,066	12,106	13,955	4,019	4,174	236,944	19,08	649,129162	97,2582964	225,5454545	94,0340909	148	40	0,53878069	19,984	6,29	3,609	2,7
47	71	17,521	26,916	16,975	12,211	13,94	5,126	3,917	178,32	15,89	345,405475	78,48896	236,4545455	87,2159091	138,5	66	0,543123084	20,008	7,023	3,861	3,519
48	65	20,375	30,466	17,317	12,179	14,104	7,664	3,571	207	17,74666667	244,536997	76,088138	216,8571429	85	33,5	57,5	0,53376153	21,081	9,45	5,725	2,906
